# Prediction of Co-Receptor Usage of HIV-1 from Genotype

**DOI:** 10.1371/journal.pcbi.1000743

**Published:** 2010-04-15

**Authors:** J. Nikolaj Dybowski, Dominik Heider, Daniel Hoffmann

**Affiliations:** Department of Bioinformatics, Center for Medical Biotechnology, University of Duisburg-Essen, Essen, Germany; Max-Planck-Institut für Informatik, Germany

## Abstract

Human Immunodeficiency Virus 1 uses for entry into host cells a receptor (CD4) and one of two co-receptors (CCR5 or CXCR4). Recently, a new class of antiretroviral drugs has entered clinical practice that specifically bind to the co-receptor CCR5, and thus inhibit virus entry. Accurate prediction of the co-receptor used by the virus in the patient is important as it allows for personalized selection of effective drugs and prognosis of disease progression. We have investigated whether it is possible to predict co-receptor usage accurately by analyzing the amino acid sequence of the main determinant of co-receptor usage, i.e., the third variable loop V3 of the gp120 protein. We developed a two-level machine learning approach that in the first level considers two different properties important for protein-protein binding derived from structural models of V3 and V3 sequences. The second level combines the two predictions of the first level. The two-level method predicts usage of CXCR4 co-receptor for new V3 sequences within seconds, with an area under the ROC curve of 0.937±0.004. Moreover, it is relatively robust against insertions and deletions, which frequently occur in V3. The approach could help clinicians to find optimal personalized treatments, and it offers new insights into the molecular basis of co-receptor usage. For instance, it quantifies the importance for co-receptor usage of a pocket that probably is responsible for binding sulfated tyrosine.

## Author Summary

Human Immunodeficiency Virus is the pathogen causing the disease AIDS. A precondition for virus entry into human cells is the contact of its glycoprotein gp120 with two cellular proteins, a receptor and a co-receptor. Depending on the viral strain, one specific co-receptor is used. The type of co-receptor used is crucial for the aggressiveness of the viral strain and the available treatment options. Hence, it is important to identify which co-receptor is used by the virus in an individual patient. Since the genome of the virus in the patient can be readily sequenced, and thus the composition of the viral proteins be determined, it could be possible to predict co-receptor usage from the viral genome sequences. To this end, we developed a method that is motivated by the insight that physical properties of gp120 will determine its specificity for a co-receptor. The method learns a computational model from structures and sequences of a crucial part of gp120, and the corresponding experimentally measured co-receptor usage. It then employs the model to predict co-receptor usage for new sequences. The high accuracy of the method could make it helpful for diagnosis and suggests that the model captures the determinants of co-receptor usage.

## Introduction

Specific protein interactions are central to biological processes, and the infection of cells with viruses is no exception there. In the case of pathogenic viruses, such protein interactions are potential targets for medical intervention. An example of particularly high relevance is Human Immunodeficiency Virus 1 (HIV-1). HIV-1 enters human cells in a process that comprises several steps, including the binding of the viral gp120 protein to the cellular receptor protein CD4 and a co-receptor protein, usually one of the two chemokine receptors CCR5 and CXCR4 [1]. The type of co-receptor used by the virus, the so-called co-receptor tropism, has a prognostic value, since patients with a CXCR4-tropic virus (“X4 virus”) progress faster to Acquired Immunodeficiency Syndrome (AIDS) compared to patients with a CCR5-tropic virus (“R5 virus”) [2]. In addition to the purely X4- and R5-tropic viruses, there are also “dual-tropic” strains, able to use both co-receptors (“R5X4 virus”). Recently, the first drug (Maraviroc [3]) that binds to CCR5, and thus inhibits productive binding of gp120, has been approved by regulatory authorities in several countries. This has made the determination of co-receptor tropism directly relevant to anti-retroviral treatment, as CCR5-inhibitors are of course inactive against X4 virus.

The standard way of determining co-receptor tropism is by cell-based assays [4], [5]. The main drawbacks of these assays are that they are currently only carried out by a handful of specialized laboratories worldwide, and that the overall procedure typically takes several weeks. These impediments to the wide application of entry inhibitors could be overcome by an approach similar to genotypic drug resistance testing [6], where drug resistance of a viral strain is inferred from comparison of mutational patterns obtained from sequencing parts of the genome of that strain with patterns of validated resistance mutations. This is a relatively fast and cheap standard procedure established in many clinics.

At first glance, genotypic testing for co-receptor tropism seems to be possible since the main molecular determinant of tropism is known to be the third variable loop (V3) of the viral glycoprotein gp120 [7], a peptide stretch of about 35 amino-acids with a disulfide bridge connecting the terminal cysteins. Unfortunately, as suggested by its name, V3 is notorious for its high sequence variability [8] including also some variability in length, and this has made it difficult to use it as a basis for genotypic co-receptor tropism testing. Nevertheless, the relevance of the quest has prompted many groups to develop models that link properties of V3 to co-receptor tropism. The importance of electrostatics for co-receptor tropism has been recognized early on, and the best-known model, the so-called 11/25-rule, refers to charges of V3-residues 11 and 25: if one of these is positive, then the virus is CXCR4-tropic [9], [10]. This rule has a specificity of more than 0.9 (few false positives), but only a low to moderate sensitivity (many false negatives) of about 0.4–0.6, depending on the test data, which is not satisfactory for routine clinical application. To improve predictions from sequence, several groups have applied machine learning methods, such as artificial neural networks [11], position specific scoring matrices [12], decision trees, or support vector machines [13]. Still, prediction accuracies fall short of what seems reasonable for regular clinical use [14]. It is unclear whether the limited accuracies are the footprint of tropism-determinants outside V3, or the consequence of model imperfections.

A milestone for the understanding of co-receptor tropism was the X-ray structure of gp120 with the V3 loop in a biological context [15]. This paved the way for the development of prediction methods that use, in addition to V3 sequence, structural information. To our knowledge, the first of these methods has been that of Sander *et al.* [16], which was mainly based on geometric distances of amino-acid pairs within the structure of V3. Although our method, detailed in the following, relies on the same experimental structure by Huang *et al.* [15], it differs from that of Sander *et al.* in several respects, e.g. it deals with indels, and, perhaps most crucially, it uses as descriptors properties that directly determine interaction of V3 with the co-receptors. By the latter we consider a seemingly trivial but fundamental fact that so far has not been thoroughly exploited: although V3 is highly variable, all X4-tropic V3 loops share *one* property, namely, they preferentially have a physical binding interaction with CXCR4, while R5-tropic V3 loops preferably interacts with CCR5. The accuracy of the method makes it attractive as clinical tool for patient tailored decisions on treatment with entry inhibitors, and it suggests that co-receptor tropism can be explained almost exclusively based on V3.

## Results/Discussion

### Overall Approach

We aim at a computational method that for a given amino acid sequence of V3 predicts the tropism class “X4” (including dual-tropics), or “R5”. Predictions by the method should have an accuracy close to 100%, and be robust against the high diversity of V3, both in terms of sequence and length.

In agreement with experimental data, we based the method on the assumption that the co-receptor tropism of HIV-1 is determined by a preferential physical interaction between a V3 loop and one of the co-receptors. We further assumed that both molecules interact while taking specific conformations. While little is known about the conformations of the extracellular parts of the co-receptors, there is a crystal structure available for a CCR5-tropic V3 loop [15]. In the first step of our approach we therefore modeled the conformations of V3 sequences of known tropism using this crystal structure as a template (see Materials and Methods). The modeled conformations enable the estimation of spatially distributed physical quantities that contribute to differential interactions of the V3 loops with the respective co-receptor, namely the values of the electrostatic potential 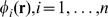 around each V3 loop 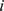 (“electrostatics hull”). Using these sets of 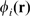 and the corresponding tropism “X4” and “R5”, respectively, we trained a first random forest [17] classifier. Tropism classification of unseen V3 sequences is performed by automated modeling of the new V3 conformation, computation of 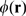, and application of the previously trained random forest. The output is a probability for the given V3 sequence to belong to the X4 class (and not to the R5 class).

Although the first step explicitly takes into account conformation dependent physical properties that are of direct relevance to the differential interaction with the two co-receptors, we do not expect a perfect classifier from this first step for a number of reasons. For example, it is unclear whether the crystal structure is an appropriate template for all V3 sequences. In fact, V3 is known to be flexible [18], and there may even be a conformational switch between X4- and R5-tropic V3 loops [19]. Hence, we trained in a second step another random forest classifier solely on V3 sequences and with the hydrophobicity scale of Kyte and Doolittle [20] as descriptor. This descriptor has been derived by amalgamating several properties of amino-acids into a single scale, notably experimental results on solubility; it happens also to map amino-acids of opposite electrical charges to different scale values. Thus, this second classifier probably captures aspects of the relation between sequence and tropism that are at least partially complementary to those considered by the first classifier.

In the final step of our approach, we trained a third random forest classifier with the two tropism class probabilities obtained from the previous two steps as input. Thus, application of the whole approach to an unseen V3 sequence includes application of a first level set of two random forests considering conformational and sequence properties, and a second level random forest using the outcomes of the first level for the final classification. Application of the classifier to a new V3 sequence to predict its co-receptor tropism takes a few seconds on a state-of-the-art CPU core. In the cross-validation, X4 sequences were detected with a sensitivity of 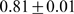 (at a specificity of 0.97), and the area under the ROC curve (AUC) was 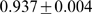 (full set of sequence and tropism data used for training and cross-validation is provided as Supporting Information). The method is described in greater detail in the following sections.

### Electrostatics Hull

The findings outlined in the introduction are compatible with a direct physical interaction between V3 and the respective co-receptor. Specifically, the 11/25 rule and the association of V3 net charge with tropism [9], [10] point to the impact of electrostatics on co-receptor tropism. In previous work, electrostatics has been considered in several ways, including the mentioned 11/25 rule, both alone and in combination with overall net charge [11], and also more complex relations such as an 11/24/25 rule [21]. Although these phenomenological rules have been helpful in guiding research, they are too simple to accurately capture the underlying molecular process, which limits their predictive power. To develop a more accurate model, we therefore first considered the one conserved feature that defines each of the tropism classes, namely the *preferential interaction* of V3 with one of the co-receptors, in particular their electrostatic interaction. Unfortunately, it is currently not possible to compute electrostatic energies of complexes of V3 and co-receptors since this necessitates availability of the structures of these complexes, which are unknown as yet. Thus, we resorted to the electrostatic potential 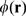 around the V3 loops as alternative descriptor. Fulfillment of the following three assumptions is sufficient, though not necessary, to justify the choice of 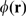 as descriptor: first, electrostatics is crucial for preferential interaction; second, the X-ray structure of the V3 loop from Huang *et al.* [15] represents the typical conformation of V3 loops, and conformations of all V3 loops can be derived as homology models from this X-ray structure; third, V3 loops bind to the co-receptors in the same binding mode. If these conditions are satisfied, preferential interactions of V3 loops with co-receptors can be mapped on differences in 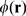, essentially because different 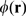 will in general lead to different interaction energies 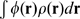 with unknown but constant co-receptor charge densities 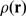.

Technically, we restricted computation of 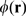 to an “electrostatics hull”, a discretized surface of 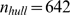 points in space around the template V3 structure of Huang *et al.* [15]. The hull should be, on one hand, wide enough to enclose all superimposed V3 loops with a certain safety margin, and, on the other hand, tight enough to reflect the differences of 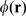 from different V3 loops. We obtained good results with a hull in a distance of 0.6 nm to the solvent accessible surface of the template V3 structure.

### Electrostatics-Based Classification

**Figure 1:**
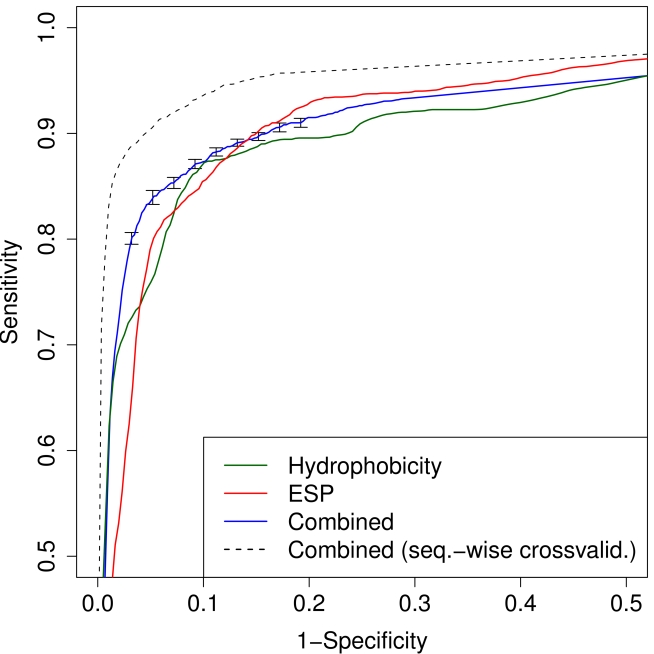
Receiver Operating Characteristic (ROC) curves of the two-level random-forest classification approach. Solid curves: averaged over ten-fold leave-one-patient-out cross-validation with random forests trained on interpolated Kyte-Doolittle hydrophobicity along normalized sequences (green), on electrostatics hull (red), and on probability outputs of the two previous random forests, i.e. second-level classification (blue); error-bars mark 95% confidence. Dashed curve: averages over ten out-of-bag predictions of second-level random forests on the full training set of sequences, disregarding that several sequences may originate from same patient.

For each V3 sequence 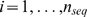 of known co-receptor tropism in the training set, a homology model was generated based on the template X-ray structure. Then the electrostatic potential 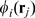 at the points 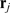 of the electrostatics hull was computed by solving the Poisson-Boltzmann Equation [22]. A random forest [17] was trained using vectors 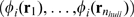 of length 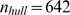 as input, and as responses the corresponding measured tropisms 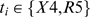, with 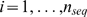. Using the leave-one-patient-out scheme for cross-validation (see “Materials and Methods”) we arrived for this classifier at an AUC of 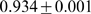 (“ESP” in Fig. 1).

**Figure 2:**
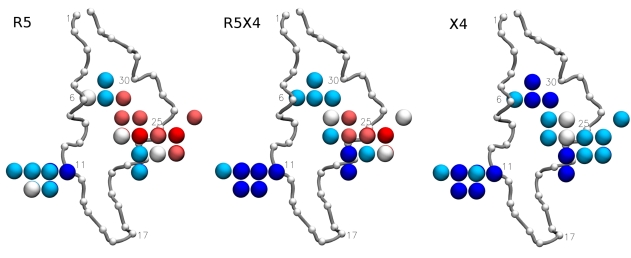
5% most important positions on electrostatics hull for tropism classification by electrostatics based random forest. The backbone of the template V3 conformation [15] is shown as tube with C_α_ atoms marked by small beads and some residues numbered for orientation, starting with the N-terminal Cys as residue 1. Points are colored according to the mean electrostatic potential 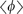 (unit 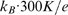) in the respective tropism class (red, 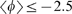; light red, 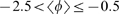; white, 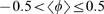; light blue, 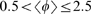; blue, 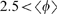).

The analysis based on the electrostatics hull opens the possibility of deriving a co-receptor specific pharmacophore pattern of V3 loops. Fig. 2 shows points of the electrostatics hull that are of highest importance for the classification by the random forest, with importance here defined as percentage decrease in accuracy in classification if, for the respective point 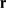 of the electrostatics hull, descriptor values 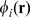 are randomly permuted [17]. As could be expected, some important points cluster in the region around residues 11, 24, and 25, though their dispersion makes it difficult to associate them with single residues. The majority of these points are located on the side to which most of the amino-acid side-chains point in the crystal structure (see also Supporting Information file Text S1). Interestingly, there is another important region on the opposite side of the loop between residues 6 and 30 that may be involved in the binding of sulfated tyrosines in the N-terminal region of CCR5 [23].

In Fig. 2 important positions are colored according to average electrostatic potential 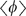 in the R5, R5X4, and X4 classes. The potential around R5-tropic V3 is generally lower as compared to X4-tropic V3, in particular around residues 24 (in agreement with the 11/24/25 rule) and 30. The coloring shows that R5X4-tropic V3 usually have 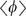 values between those of R5 and X4, while at a few patches they are chimeras of the mono-tropic classes. The latter is true between residues 6 and 30 and close to residue 25 where R5X4 on average resembles R5, and around residue 11 and close to residue 24 where R5X4 is more similar to X4.

### Hydrophobicity-Based Classification

The classification based on the values of 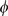 on the electrostatics hull may fail in some cases, e.g. because some V3 sequences could prefer conformations not adequately represented by the X-ray structure of Huang *et al.* [15] that forms the basis of the electrostatics hull computation. We have therefore trained a second random forest, basically using as input the Kyte-Doolittle hydrophobicity values [20] of the residues along the V3 sequences, and as response again the measured tropisms. The hydrophobicity scale seemed suitable as it also captures physically motivated properties that are relevant for binding.

An obstacle to sequence based learning was the high sequence diversity in our dataset so that standard multiple sequence alignment methods did not return clear profiles. This may have been the reason why other groups used for preparation of sequence data e.g. pairwise alignments to a reference sequence [11], manual alignments [24], or combinations of computational and manual multiple sequence alignments [12]; in these methods insertions and deletions were usually treated *ad hoc*, e.g. by removing insertions beyond a sequence length of 35. We have sought a simple algorithm that considers all sequences in a systematic and automated way irrespective of sequence length.

This algorithm essentially leads to “normalized sequences” of uniform length with interpolated hydrophobicity values as descriptors. In detail, we normalized all sequences to the maximum length of 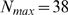 occurring in the dataset. In the normalization procedure each sequence of 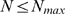 residues is first arranged along a continuous pseudo-sequence axis with equal distances of 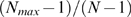 between all neighbor residues. If the first residue is placed at pseudo-sequence position 1, this equidistant arrangement brings the 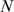
th residue to pseudo-sequence position 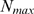, while the residues in-between are in general at non-integer positions. In the second step of the normalization procedure, hydrophobicity values at the integer positions 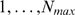 of the normalized sequence are linearly interpolated from the neighboring positions of the previously determined pseudo-sequence and their respective Kyte-Doolittle values, i.e. if the normalized sequence position 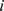 has two neighbors in the pseudo-sequence at 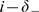 and 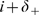 with Kyte-Doolittle values 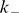 and 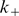, respectively, then the hydrophobicity descriptor value at normalized sequence position 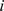 is 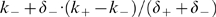. This normalization leads to uniform sequence lengths with a consistent and automated treatment of insertions and deletions.

Random forests trained on normalized sequences with interpolated Kyte-Doolittle descriptors had an AUC of 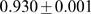, and thus about the same prediction performance in cross-validation as that trained on the electrostatics hull (see Fig. 1).

**Figure 3:**
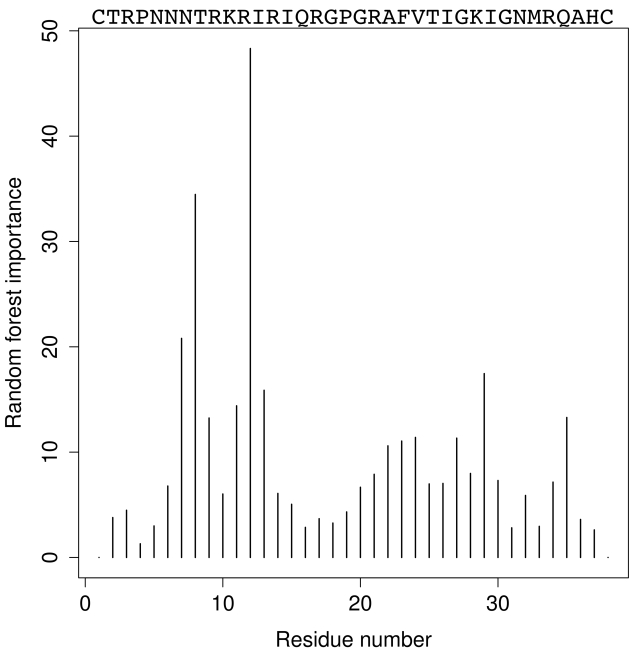
Importance of positions of normalized V3 sequence in random forest classification with Kyte-Doolittle descriptor [20]. The higher the peak at the respective position, the more important this position for correct classification of sequences with respect to co-receptor tropism. The most important region is around *normalized* sequence position 12, in agreement with the 11/25 rule. The second most important region around position 8 could be involved in binding of sulfated tyrosine on CCR5 [23]. Along the top axis, reference sequence HXB2 before normalization is given for orientation.

Fig. 3 shows the distribution of the importance for the random forest error of the normalized sequence positions 1 to 38, with importance here defined as percentage decrease in accuracy in classification if, at the respective normalized sequence position, descriptor values are randomly permuted [17]. The highest peak is in the vicinity of position 11, in agreement with the 11/25 rule (note that in the sequence normalization procedure described above most sequences are stretched towards the maximum length of 38, and this stretch shifts position 11 of the amino-acid sequence towards position 12 of the normalized sequence). Position 25 does not stick out prominently; in fact, at position 25 of the normalized sequence there is a dip in a broad hill. However, positions 22, 23, 24 and 27 in the normalized sequence have sizable importance values. The next highest peaks are around positions 8 and 29. These two positions are close in space but on opposite sides of the V3 loop in the so-called stem region (the central bulge of the V3 structure). As mentioned above, there is evidence [23] that in R5 tropic virus this region is involved in the binding of sulfated tyrosins near the N-terminus of CCR5, and that X4 and R5 tropic viruses interact differentially with these sulfated tyrosins [25].

### Second-Level Classification

**Figure 4:**
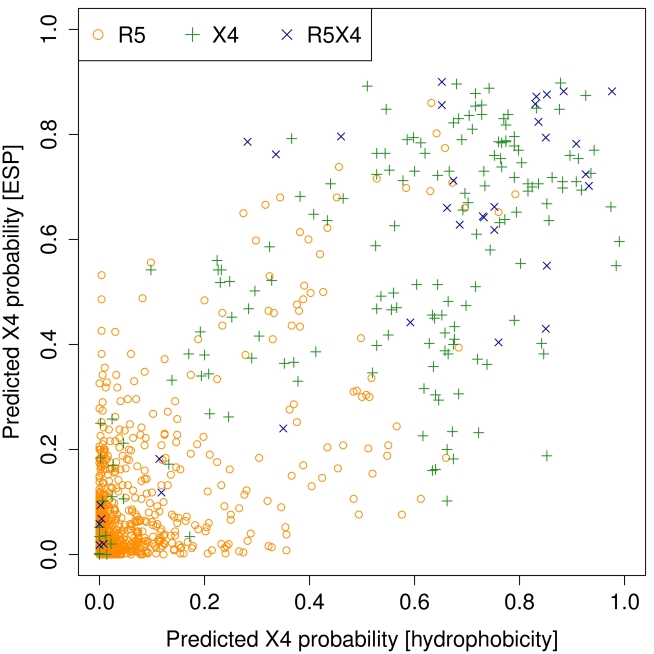
X4 class probabilities for sequences as predicted by the two first-level random forests. Vertical and horizontal axis give probabilities from electrostatics and hydrophobicity based random forests, respectively. These data points are the input for the second-level learning. Note that the sets of R5-tropic sequences (circles) and X4/R5X4-tropic (crosses) can be separated quite well in the plane spanned by the two descriptors.

In Fig. 4 the class probabilities according to the two previously described random forests are plotted for all V3 sequences in the dataset. The figure suggests that the two computational models are in part complementary, as the distribution of both tropism classes extends into the upper left and lower right quarters. More importantly for classification, the two sets of R5 and X4/R5X4 seem to be rather well separable in Fig. 4. Hence, in the spirit of “stacking” [26], we have trained another random forest for classification using the output probabilities of the electrostatics and hydrophobicity random forests as inputs and again the measured tropism classes as response.

This second-level classifier performed well (“Combined” in Fig. 1), with an AUC of 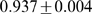 in leave-one-patient-out cross-validation. The ROC curves in Fig. 4 have several remarkable features. First, there is a striking difference between the ROC curve from sequence-wise cross-validation (dashed) and leave-one-patient-out cross-validation, with the first procedure having a clearly higher performance (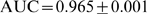). This suggests that the algorithm perceptibly takes advantage of similarities of sequences originating from the same patient. Focusing therefore on the more conservatively estimated ROC curve from patient-wise cross-validation, and on the region of low false positive rates of, say, 0.1 and less, we find that both first-level classifiers perform similarly well, and that in this region we also have the strongest added value of the second-level classification of the order of 10% in sensitivity.

The dataset used for training and cross-validation is composed of sequences from several subtypes, and we could therefore study the dependence of prediction performance of subtype. To this end we set up a contingency table of subtypes (B, C, D, other) as rows, and correct (T) and false (F) predictions in the cross-validation as columns (for example see Supporting Information file Text S1). We then carried out a 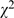-test with the null hypothesis of subtype-independence of performance, as given by the Ts and Fs. This was done for probability cutoffs between 0 and 1 in steps of 0.01 for the assignment of a sequence to the tropism class X4. It turned out that the p-value in all cases remained below 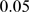, so that we should accept at this significance level the alternative hypothesis: performance depends on subtype. Specifically, the two-level random forest performs somewhat better on subtypes C and D than on subtype B (see also Supporting Information file Text S1).

Finally, one may ask whether classification with a single joint descriptor set, encompassing both electrostatics and hydrophobicity variables, could perform better than the two-level classification. Theoretical and empirical results from other groups [27], [28] suggest that second-level learning on ensembles of classifiers trained on different descriptor sets improves accuracy compared to single-level learning. A possible advantage of single-level learning with a joint descriptor set could be a consistent importance analysis across all descriptors. However, it has recently been shown that such an importance analysis in such a joint feature space is biased, and thus may be difficult to interpret [29]. Despite these caveats we tested classification with a single-level random forest with joint descriptor set, and found a performance that was good, but lower than that of the two-level approach; e.g. using the sequence-wise cross-validation the two-level approach had an AUC of 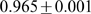, while the single-level random forest achieved an AUC of 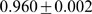, which is significantly lower (p-value of 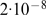 according to Wilcoxon test).

### Comparison with other Methods

For comparison with other methods we compiled an independent test set of recently published data comprising 74 sequences of various subtypes as described in the last section of “Materials and Methods”. These data are disjunct to the training set of the two-level classifier. As the data are recent, it is plausible that they were also not included in the training sets of the other machine-learning methods, though we cannot rule out this inclusion.

Apart from our two-level method, we selected for comparison the following methods: 11/25 [9], [10], 11/24/25 [21], geno2pheno [30], and wetcat [13], i.e. two simple rule-based and two machine-learning methods, the latter two via their respective web-interfaces. Since wetcat did not allow for cutoff changes, we took the specificity of 0.98, resulting from the application of wetcat to the independent test set, as reference specificity. We computed the sensitivity of the two-level approach at this specificity. Choosing a false positive rate of 1% as input parameter for geno2pheno fortunately resulted also in a specificity of 0.98, so that the sensitivities of all three machine learning methods could be compared at the same specificity. Tab. 1 shows that the two-level approach gives a higher sensitivity at this specificity than geno2pheno and wetcat. For comparison with the two rule-based methods we computed the sensitivities of the two-level approach at the specificities of these methods. Tab. 1 shows that the two-level approach has a higher sensitivity than the rule-based methods at their respective specificity, though the two simple rules do surprisingly well.

**Table 1:** Comparison with other methods on independent test set.

Method	Sensitivity	Specificity	Accuracy
geno2pheno	0.31	0.98	0.70
wetcat	0.63	0.98	0.83
two-level*^a^*	0.68	0.98	0.86
11/25	0.71	0.95	0.85
two-level*^b^*	0.73	0.95	0.86
11/24/25	0.75	0.83	0.80
two-level*^c^*	0.81	0.83	0.82

Performance of several methods on the same test set of 74 sequences with experimentally determined tropism. “Two-level” refers to the method described in this paper which is used at three different specificities, (a) at specificity of geno2pheno and wetcat, (b) at specificity of 11/25 rule, (c) at specificity of 11/24/25 rule.

The numbers in Tab. 1 should not be over-interpreted as the size of the test dataset is rather limited. For instance, on the much larger dataset used for patient-wise cross-validation of the two-level approach (provided as Supporting Information), the sensitivity of that approach was 0.762 at a specificity of 0.98, compared to the sensitivity of 0.68 on the test dataset reported in Tab. 1. Conversely, the sensitivity of the 11/25 rule decreases as we go from the smaller test set to the larger set from 0.71 (specificity 0.95) to 0.53 (specificity 0.97).

### Conclusions and Outlook

The high prediction performance suggests that tropism of the investigated sequences can be attributed almost exclusively to properties of V3, and that determinants outside V3 [31], [32] may be rare. Still, there remain a few instances of V3 sequences that were misclassified after second-level learning. For instance, there are X4 and R5X4 tropic sequences in the lower left corner of Fig. 4, i.e. both first-level classifiers, and therefore also the second-level classifier, are almost certain to see a R5 sequence, while the experimentally determined class is X4/R5X4. It is unclear whether these remaining discrepancies are due to deficiencies of our approach, tropism determinants outside the V3 loop, or experimental errors.

Further points have to be considered in view of a clinical application. First, most data for genotypic testing currently comes from bulk sequencing of blood samples that in general can contain mixtures of X4 and R5 virus. Since our method in its described form is intended for clonal sequences, the predictive performance on bulk sequencing data will be lower. Fortunately, due to the current development of “deep” sequencing [33], more and more clonal data will become available. Second, although sequences from several subtypes were present in the training set, and a first analysis of the influence of subtype was encouraging, it cannot be excluded that the performance will drop if the method is applied to subtypes that were not present in the learning set. In such cases, the method should possibly be re-trained and tested anew. Third, we have already mentioned above the occurrence of non-V3 determinants of co-receptor tropism as a possible source of errors. The frequency of such non-V3 determinants is not known, and it could be even imagined that this frequency may change over time due to a wider administration of entry inhibitors.

The presented method is based on training data comprising sequences, protein structures, and outcomes of assays. This mixture of data is available also for other cases of biological or medical interest. For instance, it would be interesting to apply the method to influenza, where structures and sequences of hemagglutinin and neuraminidase proteins responsible for contacts with host cells are available, as well as many data from immunological assays. An interesting question corresponding to prediction of co-receptor tropism in HIV could be: Is an influenza virus with a given set of protein sequences likely to infect bird, swine, or human?

## Materials and Methods

### Sequences

For training and cross-validation all V3 sequences with tropism information available from the Los Alamos HIV sequence database (http://www.hiv.lanl.gov/) were retrieved. Sequences were excluded from the analysis if they occurred with contradictory tropism annotation in the database, if they contained non-canonical amino-acid symbols, or if sequences were shorter than 30 residues. Duplicated sequences were included only once. R5X4 tropic viruses of non-clonal nature were excluded to avoid possible discordance between genotyped sequence and sequence effectively used in the phenotypical assay. These criteria led to 1151 R5 sequences, 166 X4 sequences, and 34 R5X4 sequences. 284 of these sequences contained indels (17% of R5, 51% of X4, 10% of R5X4). Most of the sequences came from subtypes B (619 R5, 81 X4/R5X4), C (218 R5, 14 X4/R5X4), and D (75 R5, 51 X4/R5X4), with the rest (239 R5, 54 X4/R5X4) spread over many different subtypes. In training and cross-validation, R5X4 sequences were assigned to the X4 class. All sequences used for training and cross-validation are provided as Supporting Information files Dataset S1 (R5), Dataset S2 (X4), and Dataset S3 (R5X4). For comparison with other methods an independent test set was collected (see below “Comparison with other methods”).

### Structures and electrostatics

V3 structures were modeled using Modeller [34], version 9.6. First, V3 sequences were subjected to pairwise alignment with the V3 sequence in the X-ray structure by Huang *et al.* [15]. Based on this alignment the structures of the V3 loops were modeled with refinement limited to optimization of side-chain positions and accommodation of insertions and deletions, if present. The 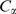 root mean square deviation of the modeled structures to the template on average was 0.085 nm with a standard deviation of 0.018 nm.

The electrostatic potential around the modeled structures was computed by solving the Poisson-Boltzmann equation with APBS [22] on a cubic grid with a spacing of 0.3 nm. PDB2PQR [35] was used to determine charges and radii. Values for the dielectric constant inside and outside V3 were scanned. Best results in the tropism prediction were achieved with a value of 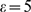 both inside and outside V3. Ionic strength was set to zero.

For the training of the machine learning model below, the values of the electrostatic potential on a hull around the modeled V3 structures was taken as input. The hull was defined as the set of grid points with minimum distance to the solvent accessible surface of the template V3 loop (solvent radius 0.14 nm) of 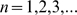 times the grid spacing distance, i.e. we tested hulls with distances of 0.3 nm, 0.6 nm, 0.9 nm, etc. to the solvent accessible surface. Best results were obtained with a distance of 0.6 nm.

### Machine Learning

Random forest analyses were carried out with the package randomForest [17] of R [36]. ROC curves were analyzed with package ROCr [37]. Cross-validation was performed in two ways. Firstly, the out-of-bag error, as provided by the random forest package was computed for the training and cross-validation set of sequences described above (alternatively, we have employed ten-fold external cross-validation but with essentially the same results). The out-of-bag error is estimated by repeatedly bootstrapping datasets, generating training sets comprising two thirds of these datasets, and predicting the remaining third [17]. Secondly, we have assessed the influence of sequence clusters originating from the same patient by a leave-one-patient-out procedure, where the random forest was trained on sequences of all patients except one, and the tropisms of the sequence or sequences of this patient were predicted; this was repeated with sequences of each patient being used as test set once. If not mentioned otherwise performance results reported in “Results/Discussion” refer to the leave-one-patient-out procedure.

AUC values of the form 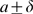 given in the text are averages over ten random forest trainings with 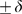 marking a 95% confidence interval estimated with a 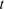-distribution.

### Comparison with other Methods

For comparative testing with other methods we collected from recent publications [38]–[41] an independent test set. All sequences from these publications were considered that did comply with the criteria applied to the training dataset described above, and, additionally were not already contained in that training set. In this way we obtained a test set of 74 sequences (43 R5, 31 X4). The test set contained sequences of subtypes B [39], AE [40], D [41], and possibly A, C, D, F, G, H, J, AE, AG, CRF11, CRF12_BF, CRF14_BG, URF from Ref. [38]. Since the last reference did not contain assignments of sequences to subtypes, and as we had to exclude some of the sequences from that reference because they were already contained in our training set, the subtypes contributed by Ref. [38] are not clear.

For the application of the 11/25 and 11/24/25 rule, sequences were pairwisely aligned with the reference V3 sequence of the HXB2 strain using Modeller [34]. HXB2 was taken from the Los Alamos sequence database.

geno2pheno and wetcat (SVM) were used via their web-interfaces at http://coreceptor.bioinf.mpi-inf.mpg.de/ and http://genomiac2.ucsd.edu:8080/wetcat/, respectively.

## Supporting Information

Text S1 Supporting information on: subtype dependence of prediction performance, statistics of multiple sequences originating from same patient, and location of important regions of electrostatics hull.
(0.69 MB PDF)

Dataset S1 All V3-loop sequences (FASTA-format) of R5-tropic virus used in the study for training and cross-validation.
(0.07 MB TXT)

Dataset S2 All V3-loop sequences (FASTA-format) of X4-tropic virus used in the study for training and cross-validation.
(0.01 MB TXT)

Dataset S3 All V3-loop sequences (FASTA-format) of R5X4-tropic (i.e. dualtropic) virus used in the study for training and cross-validation.
(2.00 KB TXT)

## References

[1] D'Souza MP, Harden VA (1996). Chemokines and HIV-1 second receptors. Confluence of two fields generates optimism in AIDS research.. Nat Med.

[2] Koot M, Keet IP, Vos AH, de Goede RE, Roos MT (1993). Prognostic value of HIV-1 syncytium-inducing phenotype for rate of CD4+ cell depletion and progression to AIDS.. Ann Intern Med.

[3] Dorr P, Westby M, Dobbs S, Griffin P, Irvine B (2005). Maraviroc (UK-427,857), a potent, orally bioavailable, and selective small-molecule inhibitor of chemokine receptor CCR5 with broad-spectrum anti-human immunodeficiency virus type 1 activity.. Antimicrob Agents Chemother.

[4] Trouplin V, Salvatori F, Cappello F, Obry V, Brelot A (2001). Determination of coreceptor usage of human immunodeficiency virus type 1 from patient plasma samples by using a recombinant phenotypic assay.. J Virol.

[5] Whitcomb JM, Huang W, Fransen S, Limoli K, Toma J (2007). Development and characterization of a novel single-cycle recombinant-virus assay to determine human immunodeficiency virus type 1 coreceptor tropism.. Antimicrob Agents Chemother.

[6] Taylor S, Jayasuriya A, Smit E (2009). Using HIV resistance tests in clinical practice.. J Antimicrob Chemother.

[7] Hwang SS, Boyle TJ, Lyerly HK, Cullen BR (1991). Identification of the envelope V3 loop as the primary determinant of cell tropism in HIV-1.. Science.

[8] Korber BT, Farber RM, Wolpert DH, Lapedes AS (1993). Covariation of mutations in the V3 loop of human immunodeficiency virus type 1 envelope protein: an information theoretic analysis.. Proc Natl Acad Sci USA.

[9] Fouchier RA, Groenink M, Kootstra NA, Tersmette M, Huisman HG (1992). Phenotype-associated sequence variation in the third variable domain of the human immunodeficiency virus type 1 gp120 molecule.. J Virol.

[10] Shioda T, Levy JA, Cheng-Mayer C (1992). Small amino acid changes in the V3 hypervariable region of gp120 can affect the T-cell-line and macrophage tropism of human immunodeficiency virus type 1.. Proc Natl Acad Sci USA.

[11] Resch W, Hoffman N, Swanstrom R (2001). Improved success of phenotype prediction of the human immunodeficiency virus type 1 from envelope variable loop 3 sequence using neural networks.. Virology.

[12] Jensen MA, Li FS, van 't Wout AB, Nickle DC, Shriner D (2003). Improved coreceptor usage prediction and genotypic monitoring of R5-to-X4 transition by motif analysis of human immunodeficiency virus type 1 env V3 loop sequences.. J Virol.

[13] Pillai S, Good B, Richman D, Corbeil J (2003). A new perspective on V3 phenotype prediction.. AIDS Res Hum Retroviruses.

[14] Low AJ, Dong W, Chan D, Sing T, Swanstrom R (2007). Current V3 genotyping algorithms are inadequate for predicting X4 co-receptor usage in clinical isolates.. AIDS.

[15] Huang CC, Tang M, Zhang MY, Majeed S, Montabana E (2005). Structure of a V3-containing HIV-1 gp120 core.. Science.

[16] Sander O, Sing T, Sommer I, Low AJ, Cheung PK (2007). Structural descriptors of gp120 V3 loop for the prediction of HIV-1 coreceptor usage.. PLoS Comput Biol.

[17] Breiman L (2001). Random forests.. Machine Learning.

[18] Stanfield R, Cabezas E, Satterthwait A, Stura E, Profy A (1999). Dual conformations for the HIV-1 gp120 V3 loop in complexes with different neutralizing Fabs.. Structure.

[19] Rosen O, Sharon M, Quadt-Akabayov SR, Anglister J (2006). Molecular switch for alternative conformations of the HIV-1 V3 region: implications for phenotype conversion.. Proc Natl Acad Sci U S A.

[20] Kyte J, Doolittle R (1982). A simple method for displaying the hydropathic character of a protein.. J Mol Biol.

[21] Cardozo T, Kimura T, Philpott S, Weiser B, Burger H (2007). Structural basis for coreceptor selectivity by the HIV type 1 V3 loop.. AIDS Res Hum Retroviruses.

[22] Baker NA, Sept D, Joseph S, Holst MJ, McCammon JA (2001). Electrostatics of nanosystems: application to microtubules and the ribosome.. Proc Natl Acad Sci U S A.

[23] Huang CC, Lam SN, Acharya P, Tang M, Xiang SH (2007). Structures of the CCR5 N terminus and of a tyrosine-sulfated antibody with HIV-1 gp120 and CD4.. Science.

[24] Lamers SL, Salemi M, McGrath MS, Fogel GB (2008). Prediction of R5, X4, and R5X4 HIV-1 coreceptor usage with evolved neural networks.. IEEE/ACM Trans Comput Biol Bioinform.

[25] Farzan M, Vasilieva N, Schnitzler CE, Chung S, Robinson J (2000). A tyrosine-sulfated peptide based on the N terminus of CCR5 interacts with a CD4-enhanced epitope of the HIV-1 gp120 envelope glycoprotein and inhibits HIV-1 entry.. J Biol Chem.

[26] Wolpert D (1992). Stacked Generalization.. Neural Networks.

[27] Zenobi G, Cunningham P (2001). Using diversity in preparing ensembles of classifiers based on different feature subsets to minimize generalization error.. volume 2167 of *Lecture Notes in Computer Science*.

[28] Nanni L, Lumini A (2009). Using ensemble of classifiers for predicting hiv protease cleavage sites in proteins.. Amino Acids.

[29] Strobl C, Boulesteix AL, Zeileis A, Hothorn T (2007). Bias in random forest variable importance measures: illustrations, sources and a solution.. BMC Bioinformatics.

[30] Sing T, Low AJ, Beerenwinkel N, Sander O, Cheung PK (2007). Predicting hiv coreceptor usage on the basis of genetic and clinical covariates.. Antivir Ther.

[31] Cho MW, Lee MK, Carney MC, Berson JF, Doms RW (1998). Identification of determinants on a dualtropic human immunodeficiency virus type 1 envelope glycoprotein that confer usage of CXCR4.. J Virol.

[32] Ghaffari G, Tuttle DL, Briggs D, Burkhardt BR, Bhatt D (2005). Complex determinants in human immunodeficiency virus type 1 envelope gp120 mediate CXCR4-dependent infection of macrophages.. J Virol.

[33] Margulies M, Egholm M, Altman WE, Attiya S, Bader JS (2005). Genome sequencing in microfabricated high-density picolitre reactors.. Nature.

[34] Šali A, Blundell TL (1993). Comparative protein modelling by satisfaction of spatial restraints.. J Mol Biol.

[35] Dolinsky TJ, Nielsen JE, McCammon JA, Baker NA (2004). PDB2PQR: an automated pipeline for the setup of Poisson-Boltzmann electrostatics calculations.. Nucleic Acids Res.

[36] R Development Core Team (2006). R: A language and environment for statistical computing.

[37] Sing T, Sander O, Beerenwinkel N, Lengauer T (2005). ROCR: visualizing classifier performance in R.. Bioinformatics.

[38] Garrido C, Roulet V, Chueca N, Poveda E, Aguilera A (2008). Evaluation of eight different bioinformatics tools to predict viral tropism in different human immunodeficiency virus type 1 subtypes.. J Clin Microbiol.

[39] Keele BF, Giorgi EE, Salazar-Gonzalez JF, Decker JM, Pham KT (2008). Identification and characterization of transmitted and early founder virus envelopes in primary hiv-1 infection.. Proc Natl Acad Sci U S A.

[40] Naganawa S, Yokoyama M, Shiino T, Suzuki T, Ishigatsubo Y (2008). Net positive charge of hiv-1 crf01_ae v3 sequence regulates viral sensitivity to humoral immunity.. PLoS ONE.

[41] Huang W, Eshleman SH, Toma J, Stawiski E, Whitcomb JM (2009). Vertical transmission of x4-tropic and dual-tropic hiv-1 in five ugandan mother-infant pairs.. AIDS.

