# Supplementary material for: Prediction of Co-Receptor Usage of HIV-1 from Genotype
Source: PLoS Comput Biol. 2010 Apr 15;6(4):e1000743. doi: 10.1371/journal.pcbi.1000743 (PMC2855328; doi:10.1371/journal.pcbi.1000743)
Supplement: Text S1 — Supporting information on: subtype dependence of prediction performance, statistics of multiple sequences originating from same patient, and location of important regions of electrostatics hull. (0.69 MB PDF) [file pcbi.1000743.s001.pdf]

Prediction of Co-Receptor Usage of HIV-1 from Genotype

– Supplementary Information –

J. Nikolaj Dybowski[1,2], Dominik Heider[1,2] Daniel Hoffmann[1,*]

1 Department of Bioinformatics, Center for Medical Biotechnology, University of Duisburg-Essen, Essen, Germany

2 These authors contributed equally

*** E-mail: daniel.hoffmann@uni-due.de**

Contents

- Tab. 1: example of a contingency table of subtypes vs. performance.
- Fig. 1: view of V3 and electrostatics hull rotated by 90% with respect to Fig. 2 of main text.
- Fig. 2: statistics over multiple sequences originating from same patient.
- Fig. 3: ROC curves for several subtypes.

Table 1. Contingency table for subtype dependence of performance of two-level classifier

Subtype	T=TP+TN	F=FP+FN
B	673	27
C	228	4
D	117	9
Other	287	6

Contingency table for a probability cutoff of 0.37 for assignment to class X4/R5X4 (this cutoff gives highest prediction accuracy T/(T+F)).

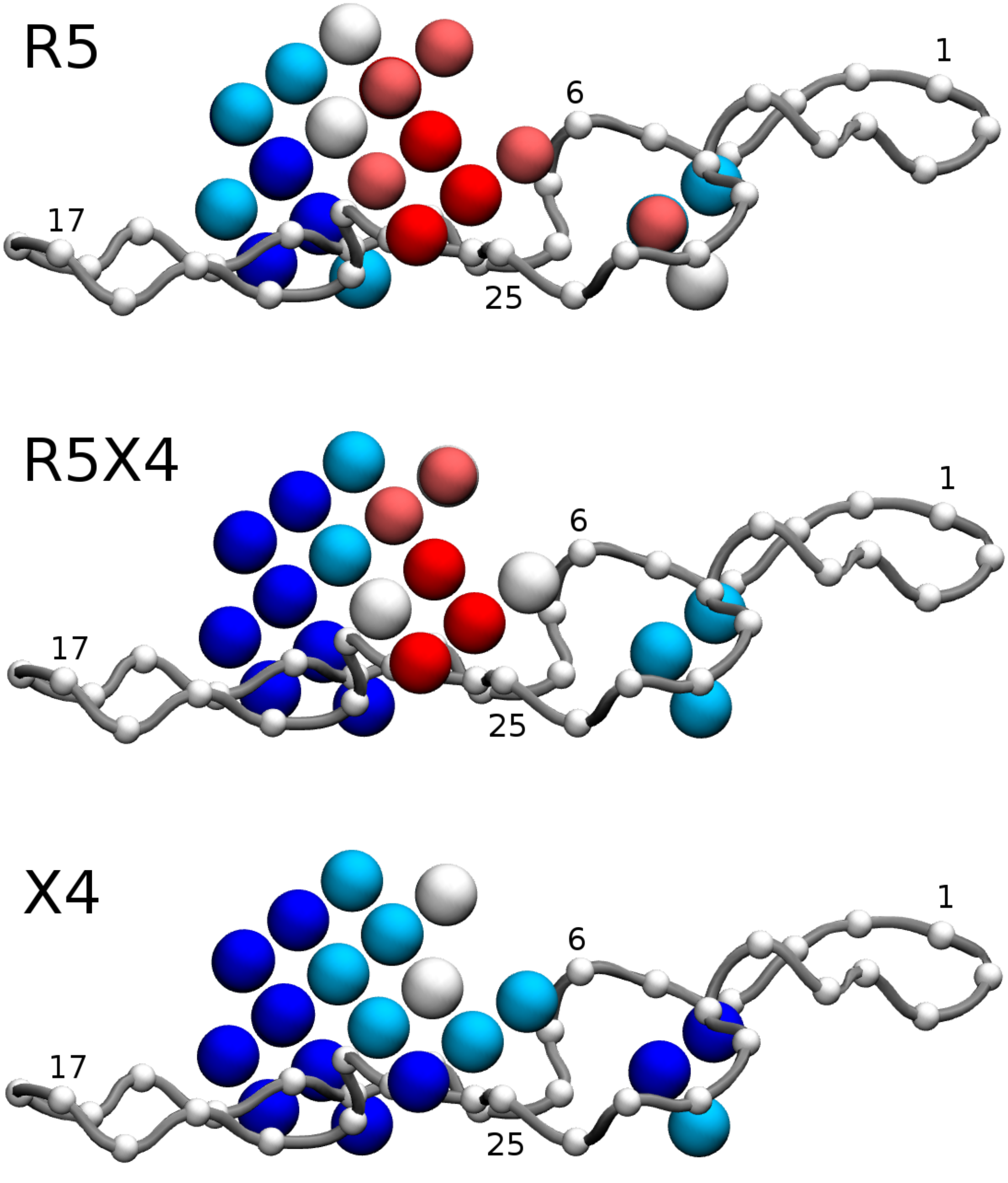

Figure 1. 5% most important positions on electrostatics hull for tropism classification by electrostatics based random forest. The backbone of the template V3 conformation is shown as tube with C_α atoms marked by small beads and some residues numbered for orientation, starting with the N-terminal Cys as residue 1. Points are colored according to the mean electrostatic potential $\langle\phi\rangle$ (unit $k_B \cdot 300K/e$) in the respective tropism class (red, $\langle\phi\rangle \leq -2.5$; light red, $-2.5 < \langle\phi\rangle \leq -0.5$; white, $-0.5 < \langle\phi\rangle \leq 0.5$; light blue, $0.5 < \langle\phi\rangle \leq 2.5$; blue, $2.5 < \langle\phi\rangle$). This view is rotated by 90° with respect to Fig. 1 of the main text.

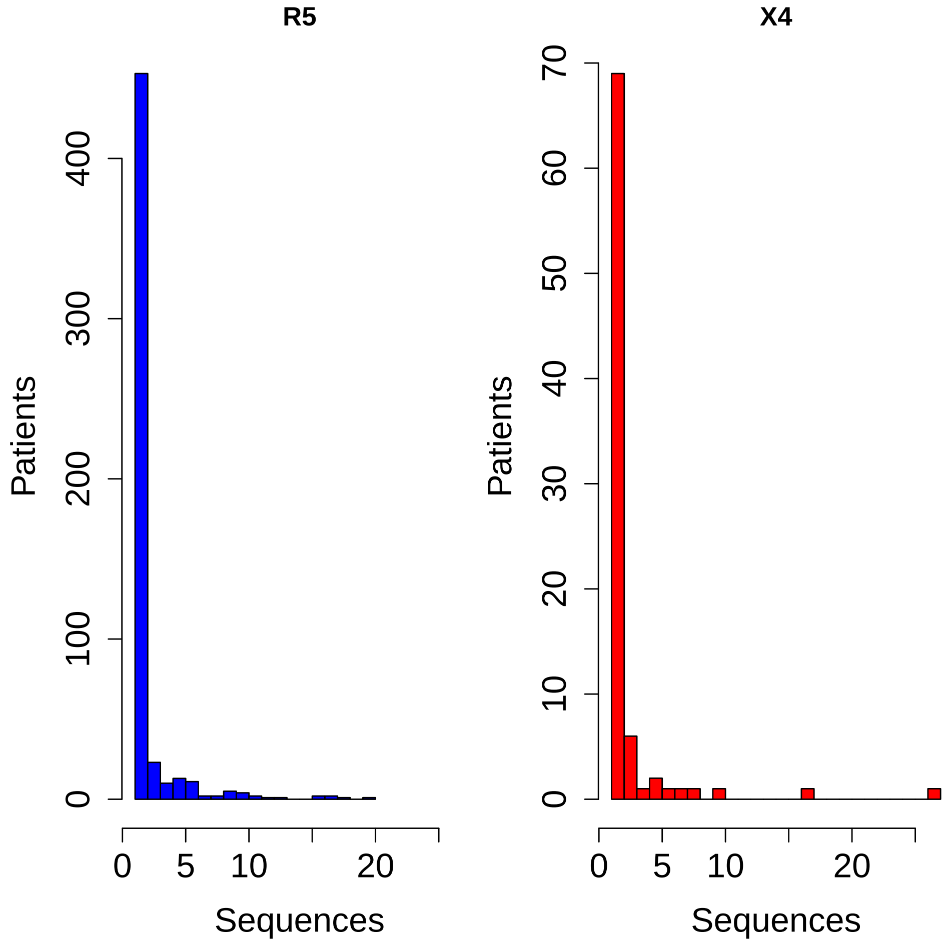

Figure 2. Distribution of patients with different numbers of sequences in the training and cross-validation set. Horizontal axis: number of sequences per patient in the dataset; vertical axis: patients with this number of sequences. Left plot: R5-tropic, right plot: X4-tropic.

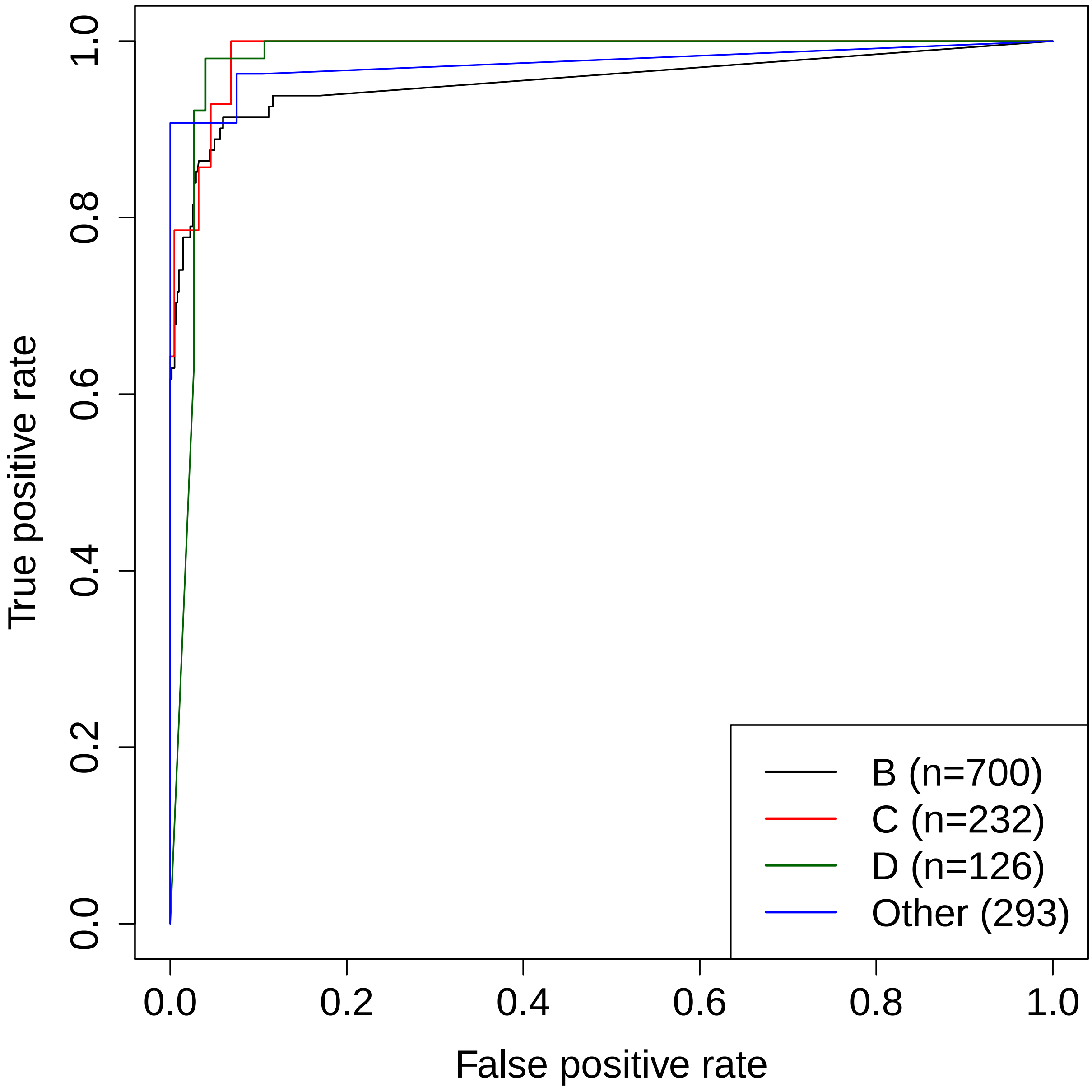

Figure 3. ROC curves for various subtypes in cross-validation data. Subtypes C, D, and other at most false positive rates are above subtype B. As described in the main text, predictive performance shows a significant dependency of subtype.
